# Chronic quadriceps tendon rupture: quadriceps tendon reconstruction using ipsilateral semitendinosus tendon graft

**DOI:** 10.1186/s13018-023-03822-5

**Published:** 2023-05-12

**Authors:** Francesco Oliva, Emanuela Marsilio, Filippo Migliorini, Nicola Maffulli

**Affiliations:** 1 Department of Musculoskeletal Disorders, Faculty of Medicine and Surgery, University of Salerno, 84084 Baronissi, Italy; 2 Department of Orthopaedic, Trauma, and Reconstructive Surgery, RWTH Aachen University Hospital, Pauwelsstraße 30, 52074 Aachen, Germany; 3 Centre for Sports and Exercise Medicine, Barts and The London School of Medicine and Dentistry, Mile End Hospital, London, E1 4DG England; 4 School of Pharmacy and Biotechnology, Keele University School of Medicine, Thornburrow Drive, Stoke on Trent, England

**Keywords:** Quadriceps, Tendon, Rupture, Chronic, Surgery, Autograft

## Abstract

Ruptures of the quadriceps tendon (QTRs) are uncommon. If the rupture is not diagnosed, chronic ruptures may develop. Re-ruptures of the quadriceps tendon are rare. Surgery is challenging because of tendon retraction, atrophy and poor quality of the remaining tissue. Multiple surgical techniques have been described. We propose a novel technique in which the quadriceps tendon is reconstructed using the ipsilateral semitendinosus tendon.

## Introduction

Quadriceps tendon ruptures (QTRs) are uncommon, affecting mainly middle-aged males (M/F = 4.2:1, mean age: 51.1 years), with an annual incidence of 1.37 patients per 100,000 persons [1]. While patellar tendon ruptures occur in younger patients (aged below 40) during sport activities, ruptures of the quadriceps tendon usually take place in older patients aged around 50–60 years following high-energy trauma [2], total knee arthroplasty [3], tumours of the knee [4] and iatrogenic and intraoperative ruptures [5, 6]. The mechanism of the trauma is often described as a sudden eccentric contraction of the quadriceps muscle group, usually to prevent a fall while climbing stairs or playing sport [7, 8]. The condition is often unilateral, although spontaneous bilateral ruptures have been reported in patients with obesity, chronic renal failure, diabetes, rheumatoid arthritis, hyperparathyroidism and gout [9–13]. Clinical findings and physical examination are the first steps to accurate diagnosis. The typical symptoms and signs are pain above the patella, a palpable gap proximal to the patella and inability to actively extend the knee [14]. Despite the clear clinical signs, the diagnosis of QTR can be missed, leading to delayed management and chronic tendon tears [10, 15]. Acute quadriceps tendon tears are classically managed with a direct repair, with transosseous sutures and anchors, even though it is still unclear which technique produces the best postoperative outcomes, given the limited number of available studies and their quality [16]. In chronic QTRs, a large substance defect or fibrotic tendon retraction can be found, and direct repair, with transosseous sutures or anchors, is not achievable [17]. In these patients, graft augmentation should be used to fill the resulting gap. Several grafts can be used, including autologous semitendinosus and gracilis tendon grafts [18, 19], peroneus longus autograft [20], iliotibial band autograft [21], local rotation flaps [22] and Achilles tendon allograft [23].

We propose a novel technique in which the quadriceps tendon is reconstructed using an ipsilateral semitendinosus tendon. This procedure has a low rate of complications and provides results that are at least comparable with those reported with other surgical approaches [7, 19, 24–26].

The technique consists of seven steps:

*Step 1*: Patient positioning.

*Step 2*: Incision.

*Step 3*: Semitendinosus tendon visualization.

*Step 4*: Harvest preparation.

*Step 5*: Tunnel drilling.

*Step 6*: Tunnel passage.

*Step 7*: Graft fixation and closure.

## Materials and methods

### Surgical technique

#### Step 1: Patient positioning

With the patient supine.

- Under general or regional anaesthesia, the leg is exsanguinated; a thigh tourniquet inflated to 300 mmHg, and the knee is prepped and draped in the usual sterile fashion.
- Make sure that the knee can be flexed to 90°.

#### Step 2: Incision

- Make a mid-line incision overlying the quadriceps tendon and the proximal patella.
- Expose the quadriceps tendon ends, free them from surrounding fibrotic adhesions and scar tissue.
- Measure the gap.

#### Step 3: Semitendinosus tendon visualization

- Through another incision over the pes anserinus, dissect the fascia and surrounding soft tissues to identify the pes anserinus.
- Incise the fascia of the pes anserinus and grab the tendon using a mosquito. The tendon may be adherent to the surrounding tissues because of the presence of vincula and cicatricial adhesion.
- Pay attention to any anatomical variants.

#### Step 4: Harvest preparation

Free the tendon of the semitendinosus from the surrounding tissues and vincula and pass it through an open tendon stripper.

**Fig. 1:**
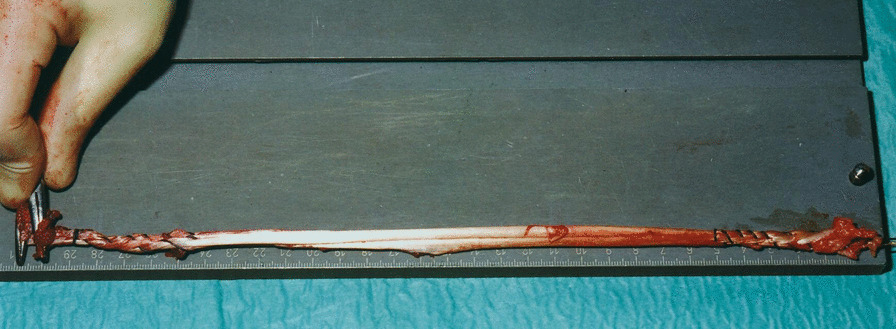
Graft preparation

- Advance the stripper proximally, and harvest it in the usual fashion.
- Prepare the proximal end in the usual fashion using five continuous two-sided Number 1 Vicryl (Ethicon, Edinburgh, Scotland) whip stitches. Detach the tendon from its insertion on the tibia and whipstitch it as described above (Fig. 1).

#### Step 5: Tunnel drilling

**Fig. 2:**
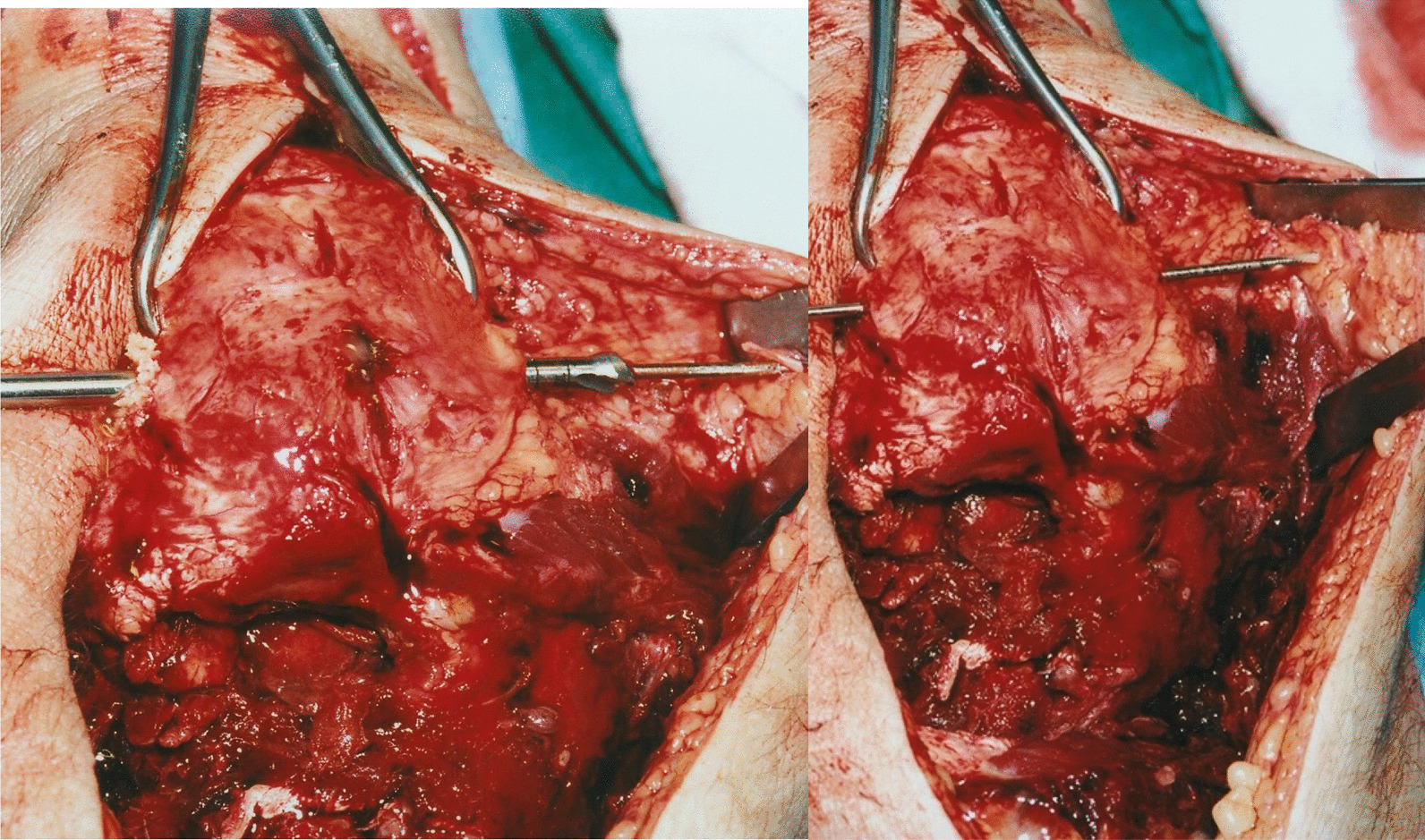
Patellar tunnel drilling

Drill a transverse tunnel through the mid-portion of the patella (Fig. 2).

- With the knee extended, after mobilization and exposure of the distal half of the patella, drill a transverse tunnel through the mid-portion of the patella with an increasing size of a cannulated burr over a Kirschner wire.
- To avoid patellar fracture, the tunnel is made with a 4.5-mm-diameter cannulated burr at first and it can be enlarged to 6 mm after, if needed. Insert a guide wire and an Ethibond Ø 0 suture (Ethicon Inc., Somerville, USA) into the tunnel from lateral to medial.

#### Step 6: Bony tunnel passage

**Fig. 3:**
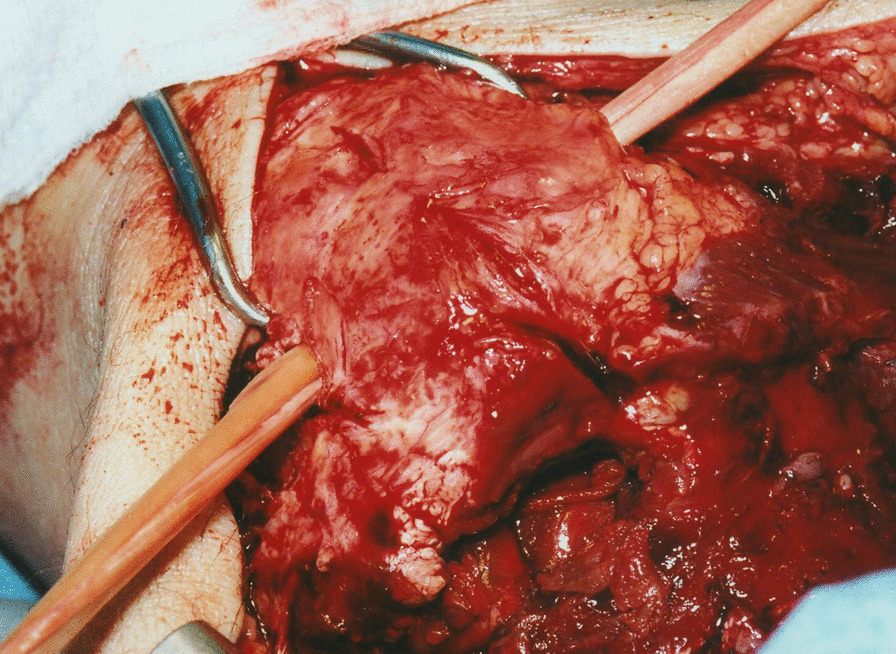
Graft passing through the patellar tunnel

**Fig. 4:**
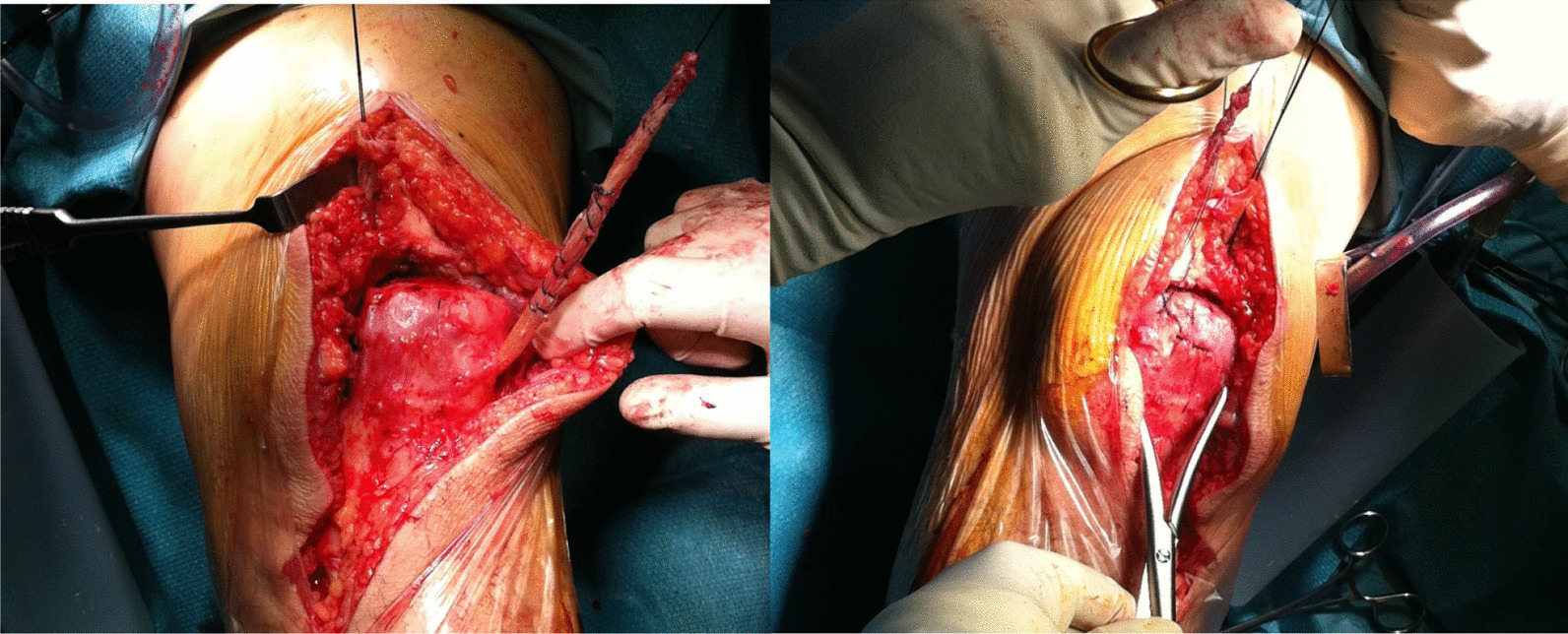
The tendon ends are passed singularly through the opposite side of the quadriceps tendon stumps

- Pass the tendon through the patellar tunnel from medial to lateral (Fig. 3).
- Cross over the tendon ends in a figure-of-eight fashion (Fig. 4).

#### Step 7: Graft fixation and closure

**Fig. 5:**
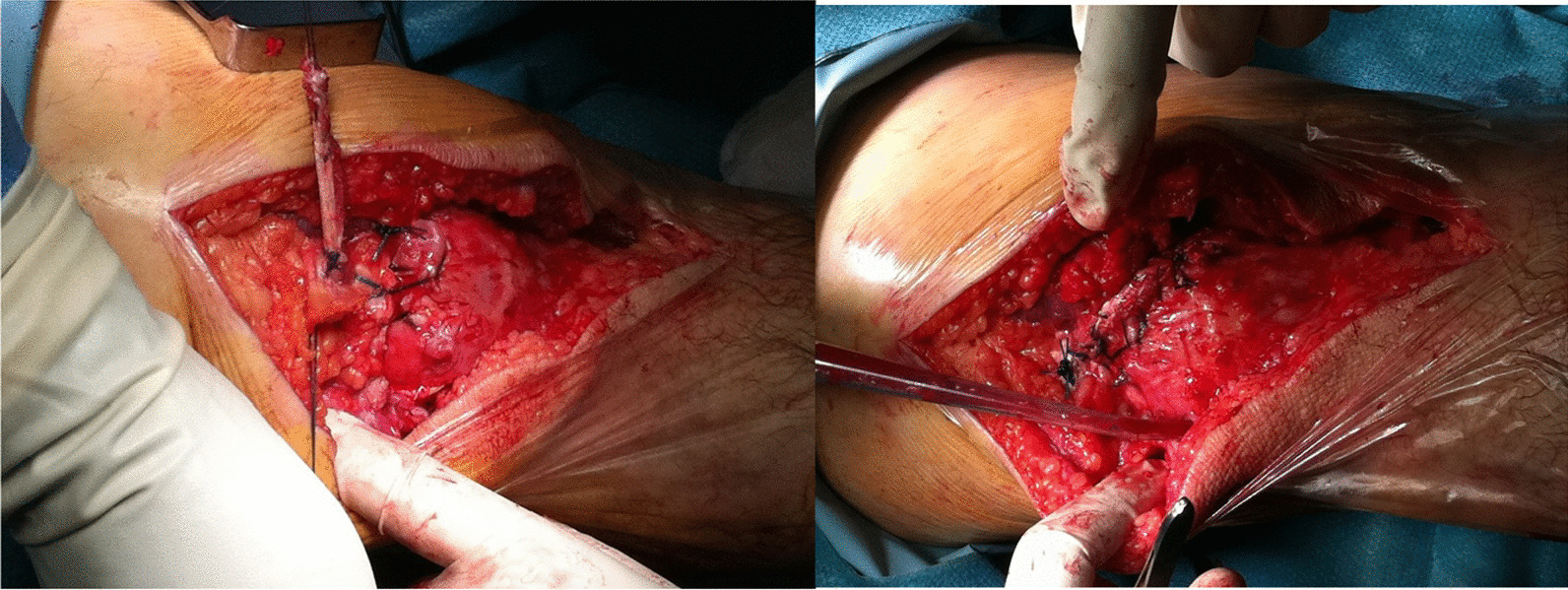
The graft is secured to the patellar exit holes and to the proximal stumps of the QT through strong absorbable sutures

- Apply distal to proximal traction to the patella to try and relocate it as close as possible to its physiological position. Since in a chronic and degenerated rupture the tendon stumps can be fragile, this passage should be done without releasing the quadriceps tendon or further dissect the peri-patellar tissues, to avoid further substance loss.
- Secure the graft suturing the tendon stumps to the periosteum of the patellar tunnel exit holes with strong absorbable sutures. Secure the free tendon ends to the proximal retracted stump of the torn quadriceps tendon (Fig. 5).
- Juxtapose the subcutaneous fat using fine absorbable sutures, close the skin with subcuticular absorbable sutures.
- The leg is immobilized in full extension using a cylinder cast or using a commercially available splint in extension leaving the ankle free.

## Discussion

Quadriceps tendon tears are the most frequent injury of the extensor mechanism of the knee, after patellar fractures [1]. The integrity of the quadriceps tendon and the whole extensor mechanism guarantees extension movements, gait, jumping, sport activities and the normal function of the lower limb [27]. Reito et al. reported an increasing incidence of QTR, mostly in patients aged 50 years or more, therefore more likely to present comorbidities [28]. Contrary to other tendon ruptures, QTRs occur more often after a fall or high-energy traumas rather than during sport activities [29]. At physical examination, surgeons should distinguish between a real extension lag and limitation of motion caused by pain [30]. For this reason, examination of the contralateral limb is crucial [19]. Different anatomical sites of the tendon can be affected. A QTR can occur at the tendon–bone junction, or 1–2 cm from the superior pole of the patella, a hypovascularized area of the tendon [31]. There are few evidence and randomized controlled trials on the optimal surgical management of chronic quadriceps tendon ruptures in the current literature [15]. Recently, Elattar et al. overviewed the management of chronic QTRs and offered a treatment algorithm based merely on the timing of diagnosis and surgery [17]. Indeed, early diagnosis allows to achieve better treatment and outcome and also active/passive ROM recovery [32]. As previously stated, there are different surgical strategies for acute QTRs, including transosseous patellar tunnels, end-to-end sutures, anchor fixation and graft augmentation [16, 33]. In the case of chronic QTR with loss of substance, the use of an autologous graft is advisable to restore the anatomy and function of the quadriceps tendon [30]. McCormick et al. performed a semitendinosus and gracilis autograft for revision of chronic QTR to restore large tendon defects. The hamstring tendon graft was weaved through the QT and passed through three transosseous patellar tunnels, then tensioned and secured to the inferior pole of the patella [18]. Ayas et al. treated QT retraction following a non-union patellar fracture with a peroneus longus autograft. A peroneus longus autograft was harvested and split longitudinally and then passed through the tunnels produced in the tibia and patella. The ends of the grafts were sutured to the quadriceps tendon proximally and the patellar tendon distally with the knee flexed at 45° [20]. Auregan et al. presented a case of quadriceps tendon re-rupture after TKA treated with a hemisoleus rotation flap that was divided into two equal flaps and, with an osteotome, a medial calcaneal bone block was harvested. The soleus and gastrocnemius were separated from each other in a proximal to distal direction obtaining a composite graft. This graft was passed from the posterior to the anterior compartment through a subcutaneous incision. The flap was then passed through a slit in the quadriceps tendon and in a 2-mm patellar tunnel and sutured [22]. Recently, Danaher et al. confirmed that reconstruction using a graft should be the standard of care in chronic tears and mid-substance injuries of the whole extensor apparatus of the knee. Furthermore, when dealing with compromised and degenerated tendon tissue, collagen patches may further improve quadriceps and patellar tendon healing [34].

The technique described in this report is simple and effective, using the semitendinosus tendon which is frequently harvested for soft tissue reconstruction around the knee and leaves the patient with no disability. Therefore, this technique can be used for quadriceps tendon reconstruction after:

- chronic ruptures with loss of substance (> 2 cm),
- failure of previous repair,
- chronic ruptures (> 6 weeks),
- all the instances when the tendon ends cannot be juxtaposed.

## Conclusion

Surgical treatment of chronic QTR is challenging and lacks evidence-based guidelines. We propose the use of ipsilateral semitendinosus tendon autograft as an applicable quadriceps tendon reconstruction surgical technique.

## Data Availability

The datasets generated and/or analysed during the current study are available throughout the manuscript.
